# Life Satisfaction Development in the Transition to Adulthood: Differences by Gender and Immigrant Background

**DOI:** 10.1007/s10964-021-01560-7

**Published:** 2022-01-13

**Authors:** Juul H. D. Henkens, Matthijs Kalmijn, Helga A. G. de Valk

**Affiliations:** Netherlands Interdisciplinary Demographic Institute-KNAW, University of Groningen, The Hague, The Netherlands

**Keywords:** Life satisfaction, Development, Transition to adulthood, Gender, Children of immigrants

## Abstract

Life satisfaction is crucial for healthy development into adulthood. However, it is yet largely unknown how life satisfaction develops in the transition to adulthood. This study examined life satisfaction development in this transition and paid special attention to differences between boys, girls, children of immigrants, and nonimmigrants. Unique longitudinal data of seven waves (2010–2018) of the Children of Immigrants Longitudinal Survey Germany were used. Respondents (*N* = 3757, 54% girls, 78% nonimmigrants, M_age weighted_ = 14.6, *SD* = 0.6 at wave 1) were followed between ages 14 and 23 and multi-level random effect models were applied. Life satisfaction developed in a nonlinear way in the transition to adulthood (M-shape), with overall decreases between age 17 and 18 and between age 20 and 23. Girls reported lower life satisfaction levels in adolescence and more unstable trajectories than boys, where girls with immigrant backgrounds represented the least advantageous life satisfaction trajectory. Differences in life satisfaction between groups decreased from age 19 onwards.

## Introduction

Life satisfaction is key for a positive transition from adolescence to adulthood (Hawkins et al., 2009; Proctor et al., 2009). This transition is a developmentally dense period (Switek 2014) in which young people^1^ often experience major life events in multiple life domains, from physical maturation and identity development to leaving the parental home, starting intimate relationships, finishing education, and entering the labor market (Buchmann & Kriesi, 2011). Since life satisfaction can be influenced by life events (Ash & Huebner, 2001; Fujita & Diener, 2005), life satisfaction is likely to change in this turbulent life phase (Lucas & Donnellan, 2007). However, so far little is known about how life satisfaction evolves in the transition and what can be critical periods and turning points. In addition, in increasingly diverse societies it is essential to understand whether this development differs by gender and immigrant background. Because of gender differences in adolescent development, it is likely that life satisfaction develops differently for boys and girls. Additionally, the share of people with an immigrant background is increasing in European societies, but it is unknown whether life satisfaction development is similar for children of immigrants (i.e., young people who were born in the country of residence but had at least one foreign-born parent) and nonimmigrants. Existing literature has either studied life satisfaction development for the majority group only or compared levels of life satisfaction at only one point in time. Longitudinal research in boys and girls of immigrant and nonimmigrant backgrounds is needed to understand potential differences in trajectories and key turning points in life satisfaction early in the life course. Studying trajectories will also highlight whether one-time measures are a good proxy for life satisfaction measurement or whether cross-sectional measures at different ages may over- or underestimate life satisfaction. Since experiences in early life are known to determine later life chances, these insights are crucial to create equal opportunities with long-lasting effects on individuals’ lives. More insight into developmental trajectories of life satisfaction for different subgroups can help to optimally time interventions in order to foster positive development into adulthood for each subgroup separately and can reveal subgroups at risk of lower life satisfaction. With unique new longitudinal data (Children of Immigrants Longitudinal Survey Germany [CILS-4EU-DE] Kalter et al., 2019) this study can overcome cross-sectional limitations in previous research and study life satisfaction development in the transition to adulthood (between ages 14 and 23) among young people of diverse origins living in Germany.

### Life Satisfaction Development

Life satisfaction is defined as a subjective measure of global quality of life based on one’s own set of criteria (Shin & Johnson, 1978) and thus focuses on an individual’s self-report rather than on objective life conditions (Dew & Huebner, 1994). Life satisfaction is not a static state but may develop over time (Lucas & Donnellan, 2007). This may be even more the case in the transition to adulthood, where young people are experiencing many transitions in a short period of time. Several developmental theories emphasize the turbulence that comes with the transition from adolescence to adulthood. Erikson characterized adolescence as the period of identity formation and role confusion (Erikson, 1968). It is in adolescence that rapid physical growth, hormonal changes, increased sexual activity, and identity and autonomy development co-occur with an increase in negative moods (Steinberg & Silk, 2002). Moreover, in the pursuit of autonomy, there is temporarily more conflict and less closeness between parents and their adolescent children (Aquilino, 1997; De Goede et al., 2009). Arnett (2000) focused on the period between adolescence and adulthood, so-called emerging adulthood, highlighting that young people left the dependency of adolescence without fully entering adulthood. Emerging adulthood is characterized by a strong focus on independence and autonomy development, exploration of adult roles, and many major life events in a relatively short period (e.g., leaving the parental home, school to work transition, union formation). As such, Sameroff (2010) characterized the transition to adulthood as a period of qualitative change and discontinuity in his unified theory of development. Finding your way in this “jungle” of changes can be stressful, confusing, or disappointing (Arnett, 2000) and as such lead to lower life satisfaction. However, successfully navigating through the transition and reaching the new developmental stage of adulthood (i.e., becoming independent) may increase life satisfaction (Arnett, 2007; Schulenberg et al., 2004).

Previous studies looking at short trajectories showed life satisfaction to decrease over the course of adolescence. Some found a linear decrease in adolescence (Goldbeck et al., 2007 for ages 11 to 16; Shek & Li, 2016 for age 12 to 15), others found the negative trend to slow down into emerging adulthood (Burger & Samuel, 2017 for ages 15 to 20; Orben et al., 2020 for ages 10 to 24), or found the decrease to start in late adolescence (Willroth et al., 2021 for ages 14 to 21). In contrast, Finnish studies reported life satisfaction to increase (Salmela-Aro & Tuominen-Soini, 2010; Salmela-Aro & Tynkkynen, 2010 for ages 15 to 17). Because of the expected fluctuations in life satisfaction, inconsistencies in findings might be partially due to different age ranges studied in previous research. Furthermore, these earlier studies were conducted in different countries and may therefore highlight potential heterogeneity in life satisfaction development across cultures, which could be linked to a culturally dependent transition into adulthood (Arnett, 2007; De Valk, 2006). For example, the country-specific school transition in Finland might account for deviating results in Finnish research (Salmela-Aro & Tuominen-Soini, 2010; Salmela-Aro & Tynkkynen, 2010). Similarly, trajectories of minority groups (Willroth et al., 2021) may not be generalizable to majority populations. The present study among a large population sample, including a wide age range with yearly intervals from adolescence to adulthood, is a unique contribution to the understanding of normative trajectories of life satisfaction in a Western European context.

Studies on well-being measures that are related to life satisfaction (e.g., self-esteem) reported an increase in early adulthood (Galambos et al., 2006; Gestsdottir et al., 2015), and thus it could be hypothesized that life satisfaction increases accordingly. However, since the existing literature on life satisfaction development is mostly focused on either adolescence (approximately ages 11 to 17) or emerging adulthood (approximately ages 18 to 25) and longitudinal research that includes the transition from adolescence to adulthood is limited, it is unknown from what age onward an increase in life satisfaction can be expected. In the German context of this study, like in most European societies, young people legally become adults from the age of 18. This strongly institutionalized age norm of adulthood (see also Billari & Liefbroer, 2010) is a key marker in the transition to adulthood that comes with new (also legal) rights and obligations for all young people living in Germany. This new independence, combined with adult rights and responsibilities, may result in anxiety over the new roles. As such, in anticipation of this moment, turning 18 can go hand in hand with lower levels of life satisfaction while life satisfaction may increase in the years afterward when the new position is more stabilized and has become the “new normal”.

### Gender Differences

Studies have extensively shown that adolescent development is different for boys and girls (for a review, see Perry & Pauletti, 2011). The accelerated maturation hypothesis suggests that, because girls start puberty earlier than boys (Negriff & Susman, 2011), the expected decrease of life satisfaction in adolescence might start earlier in girls than in boys (Orben et al., 2020). In addition, girls report more, and are also more sensitive to, interpersonal stressors (such as family or friendship-related conflicts) than boys (Marcotte et al., 2002; Oldehinkel & Bouma, 2011). Under the influence of hormonal changes in adolescence (Schulz & Sisk, 2016), this gender difference is particularly salient in adolescence (Rudolph & Hammen, 1999). Because stressful events can negatively influence life satisfaction (Fujita & Diener, 2005), girls may experience a steeper fall in life satisfaction in adolescence than boys. However, after adolescence, a new hormonal equilibrium will be reached. The gap between boys and girls is therefore likely to diminish after adolescence.

Previous research corroborates the accelerated maturation hypothesis and stress-sensitivity perspective. Girls’ life satisfaction decreased from early adolescence and stabilized or even increased from late adolescence into emerging adulthood (Keresteš & Štulhofer, 2020; Orben et al., 2020). Although also boys’ life satisfaction decreased, it set off in emerging adulthood, years later than found in girls. This resulted in lower life satisfaction levels for males than for females in adulthood (Keresteš & Štulhofer, 2020; Orben et al., 2020). In line with this, research that followed middle to late adolescents found that girls’ life satisfaction started lower than that of boys, but it increased, whereas boys showed high and stable life satisfaction trajectories (Salmela-Aro & Tynkkynen, 2010). These findings could imply that boys did not yet experience a decrease in life satisfaction in adolescence. The different timing of the life satisfaction decrease in adolescence may partly explain why adolescent research consistently found girls to report lower life satisfaction (De Looze et al., 2020; Moksnes & Espnes, 2013), whereas in adulthood females score higher (Gestsdottir et al., 2015; Lysberg et al., 2018). Therefore, a longer observation window to study the trajectories of life satisfaction is needed to understand the potentially gendered patterns.

### Children of Immigrants and Nonimmigrants

In Germany, like in most European countries, the share of people with an immigrant background is taking up to a quarter of the population (Statistisches Bundesamt, 2021). Children of immigrants in Germany grow up in two potentially different cultures: The often more collectivistic origin culture of the parents (by far the largest group originates from Turkey), with more focus on interdependence and relatedness (Idema & Phalet, 2007; Nauck, 2001) versus a more individualistic host culture, where the focus is more on independence (Arnett, 2007). According to the acculturation gap distress theory (Szapocznik & Kurtines, 1993; Lui, 2015) growing up in two different cultures could be stressful. That is, the difference might lead to a cultural mismatch in important life values, acculturation gaps between parents and children, divergent expectations, and parent–child conflict, which can result in lower life satisfaction levels for children of immigrants compared to nonimmigrants (Lui, 2015). In the phase of emerging adulthood, the societal focus of individualistic societies is on identity and autonomy development (Arnett, 2007). It is therefore likely that the cultural mismatch between more individualistic and collectivistic cultures becomes particularly salient in emerging adulthood for children of immigrants, which may then relate to a decrease in life satisfaction.

In addition, life satisfaction is expected to change due to developments and life events typical for the transition to adulthood. The timing of these typical developments and life events may differ between children of immigrants from more traditional cultures and nonimmigrants (e.g., pursue autonomy and leaving the parental home later, but marry earlier, De Valk, 2006; Kwak, 2003). Therefore the timing of changes in life satisfaction over time might differ between children of immigrants and nonimmigrants.

Empirical research so far has focused on differences in *levels* rather than on the *development* of life satisfaction between children of immigrants and nonimmigrants. Results are inconsistent; Some research found children of immigrants to have lower levels of life satisfaction (Stevens et al., 2015), whereas others found no significant differences (Rodríguez et al., 2020), or found children of immigrants to have higher life satisfaction (Tang, 2019). Since these studies measured life satisfaction at one point in time, these inconsistencies may indeed indicate differential development for children of immigrants and nonimmigrants. The current longitudinal study into differences in life satisfaction development can help to understand these inconsistencies.

### Double Disadvantage

The double disadvantage hypothesis (King, 1988) highlights the potential accumulated negative effects of belonging to multiple social groups at risk of lower life satisfaction, such as having an immigrant background and being female. In addition, while in individualistic cultures, boys and girls follow equal pathways into adulthood, this transition is more gendered in collectivistic cultures (De Valk, 2006). Gendered socialization in immigrant families is commonly marked by more restrictiveness and monitoring for girls than for boys, which might not align with equal gender roles in the host country (Dion & Dion, 2001). This can have detrimental effects on well-being especially for girls with immigrant backgrounds (Céspedes & Huey, 2008; Talbani & Hasanali, 2000). Recent cross-sectional research found immigrant girls to have lower levels of life satisfaction than non-immigrant girls, but also than immigrant boys, indicating a double disadvantage of being a girl and having an immigrant background, at least with respect to a subjective outcome such as life satisfaction (Kern et al., 2020).

### German Context

The educational career of students in Germany is tracked into different levels at the age of 10/11 (Kristen & Granato, 2007). Those in vocational tracks are attending school until age 16, those in academic tracks until age 19. At the age of 18, young people are legally adults including full legal capacity, right to vote, ability to drive, drink alcohol, smoke, and determine their own place of residence. The current population of German people has a diverse background; in 2021, more than a quarter of the German population had a first- or second-generation immigration background (Statistisches Bundesamt, 2021). Among those below the age of 25, even a third has at least one parent born abroad (DJI, 2020). This diverse population results from past international migration patterns when different origin groups arrived in the country for different motives (Kalter & Granato, 2007; Van Mol & De Valk, 2016): Turks, Italians, and Former Yugoslavians who mainly came as labor migrants from the 1960s onwards, those from the Former Soviet Union and Poland with German origin who came back in the aftermath of the Iron Curtain fall (“Aussiedler”), those who flew war in former Yugoslavia in the 1990s, and more recently, labor migrants from Eastern European countries, in particular Poland (DJI, 2020). Hence a large share of children of immigrants in this study, in line with the largest migrant populations in the country, have parents born in Turkey, Poland, the Former Soviet Union, Former Yugoslavia, and Italy.

## Current Study

Life satisfaction is crucial for a healthy transition to adulthood but so far, little is known about how life satisfaction develops in the transition from adolescence to adulthood, and whether this development differs between boys and girls and between children of immigrants and nonimmigrants. The current study uses longitudinal data covering the ages 14 to 23 to examine life satisfaction development in the transition into adulthood for boys and girls and children of immigrants and nonimmigrants separately. Children of immigrants refer to those born in Germany with one or two foreign-born parents (also referred to as those with immigrant backgrounds). Data from the first seven waves (2010–2018) of the Children of Immigrants Longitudinal Survey conducted in Germany (CILS-4EU-DE; Kalter et al., 2019) were used. A systematic, school-based overrepresentation of children of immigrants allowed studying life satisfaction development for young people with and without an immigrant background separately. First, based on developmental theory, a U-shaped development of life satisfaction was expected, with decreasing life satisfaction over the course of adolescence, followed by an increase in emerging adulthood. Second, following the accelerated maturation hypothesis and stress-sensitivity perspective, gendered life satisfaction trajectories were expected. Specifically, it was hypothesized that girls have lower starting levels of life satisfaction in adolescence, but that gender differences decrease into emerging adulthood. Third, by using a cultural mismatch perspective, it was hypothesized that children of immigrants have a steeper decrease and different timing of life satisfaction development than nonimmigrants. Finally, based on the double disadvantage perspective, it was expected that girls with immigrant backgrounds would have the lowest trajectories of life satisfaction. Because children of immigrants were coming from different origins, trajectories for the five largest immigrant groups in this study (i.e., from Turkey, Poland, Former Soviet Union, Former Yugoslavia, and Italy) were also studied separately. These additional subgroup analyses were more explorative due to sample size restrictions.

## Methods

### Participants and Procedure

For this study, German data from the first seven waves of CILS4EU-DE (Kalter et al., 2019) were used. Students were sampled with a school-based, stratified three-stage sample design. Students filled out questionnaires in their school class in the first three waves (2010–2013) and were followed with self-report questionnaires up to 2018, resulting in seven waves (waves 6 and 7 were two years apart). Parental consent was required for participation. Participants who could not be interviewed in school were approached individually and interviewed via phone, post, or web. Schools with high proportions of immigrant students were oversampled. This means that the nonimmigrant population in this study went to schools with relatively high immigrant proportions. To account for this, sample design weights were used (for more details about sampling and weights, see CILS4EU-DE, 2019).

Observations were excluded of those who participated in only one wave (i.e., no development, *n*_unweighted_ = 801), participants younger than 14 or older than 16 at wave 1 (*n*_unweighted_ = 680), with missing data on gender (*n*_unweighted_ = 9), and who were first-generation immigrants (*n*_unweighted_ = 1844). For first-generation immigrants, multiple factors that are potentially linked to the international migration process are at play, which may influence life satisfaction. In addition, first-generation immigrants were significantly older in the first wave, which may suggest a different age pattern of life satisfaction. Of the analytical sample, 40% participated in all seven waves. Attrition analyses (see Appendix A1, Table A1) indicated that the likelihood to drop out increased with age (*b* = 0.18, *SE* = 0.02, *p* < 0.001). Importantly, life satisfaction in the previous wave was a weak but significant positive predictor of dropout in the next wave for boys with immigrant backgrounds (*b* = 0.13, *SE* = 0.06, *p* = 0.022). However, the magnitude of this effect was small, so that attrition bias was likely to be limited. To keep the sample as large as possible, and to avoid selectivity, all participants with data on at least two waves were included. Missing data on the dependent variable were not imputed. This resulted in a final weighted sample of 3757 participants (M_age_ = 14.6, *SD* = 0.6 at wave 1) and 19,041 observations (person-years) for the main analyses. Weighted sample statistics are presented in Table 1.

**Table 1:** Weighted sample statistics per age

Age	14	15	16	17	18	19	20	21	22	23	Total
Boys^a^	819	1752	1658	1567	1348	1162	842	429	626	161	10364
(%)	45.29	49.28	51.15	47.43	46.22	47.70	46.71	42.24	45.16	53.87	47.62
Girls	989	1803	1583	1736	1569	1274	960	586	760	138	10345
(%)	54.71	50.72	48.85	52.57	53.78	52.30	53.29	57.76	54.84	46.13	53.64
Nonimmigrants	1442	2737	2487	2564	2285	1903	1433	803	1085	219	16957
(%)	79.79	76.98	76.73	77.62	78.33	78.11	79.48	79.08	78.29	72.96	77.91
Children of immigrants	365	819	754	739	632	533	370	212	301	81	4807
(%)	20.21	23.02	23.27	22.38	21.67	21.89	20.52	20.92	21.71	27.04	22.09
Total^b^	1808	3555	3241	3303	2917	2436	1802	1078	1386	299	19286
	100.00	100.00	100.00	100.00	100.00	100.00	100.00	100.00	100.00	100.00	100.00
Life satisfaction Mean (*SD*)	7.58 (2.10)	7.51 (2.16)	7.69 (1.97)	7.70 (1.85)	7.46 (1.98)	7.64 (1.86)	7.83 (1.57)	7.53 (1.83)	7.41 (1.86)	7.11 (2.13)	7.58 (1.35)

*Note*. *N*’s are based on the sample analyzed in multilevel models (i.e., including filters for age, gender, immigrant background, and missing life satisfaction values)

^a^All weighted subsample sized are rounded, totals are based on rounded *n*’s. Mean ages are presented per wave

^b^Totals are based on weighted numbers of boys and girls

### Measures

#### Life satisfaction

The dependent variable life satisfaction was assessed the same way in all seven waves by the single-item Cantril ladder: “On a scale from 1 to 10, how satisfied are you with your life in general?”. The Cantril ladder is repeatedly validated in samples of young people (Jovanović, 2016; Jovanović & Lazić, 2018; Levin & Currie, 2014). The overall mean life satisfaction of all ages was 7.56 with a standard deviation of 1.51 (the latter being important for evaluating the magnitude of the effects).

#### Demographics

Age was constructed with month and year of birth and was rounded such that 12–12.99 was 12 years old. Age was rescaled such that x = 0 represented age 14.^2^ Gender was coded as 0 (boy) and 1 (girl). Immigrant background was constructed with participant’s country of birth and country of birth of father and mother (Dollmann et al., 2014). Participants born in Germany with at least one parent born outside Germany were considered children of immigrants (1). Participants born in Germany, with both parents born in Germany were coded as of nonimmigrant origin (0).

### Analyses

Data were prepared and analyzed in Stata version 16.1 (StataCorp, 2019). Because this study examined the development of life satisfaction over time and thus by age of the respondents, the person-period file was organized by age and not by wave.

Following a stepwise procedure, random-effects models were estimated where age-specific observations were nested in students. The models were estimated with Maximum Likelihood. First, an empty model (Model 0) was estimated to examine whether observations within persons were more alike than between persons (i.e., whether data was nested). In Model 1, age was added (as a continuous variable in Model 1a, as an indicator variable in Model 1b) to examine the development of life satisfaction over time. In Model 2a and 2b, gender and immigrant background were added separately and in Model 2c simultaneously to examine whether life satisfaction levels differed between boys and girls and between children of immigrants and nonimmigrants. In Model 2d, the interaction gender*immigrant background was added to investigate whether the effect of immigrant background differed between boys and girls.

To examine whether life satisfaction trajectories differed across groups, four models were tested. Model 3a included a cross-level interaction age*gender, to examine whether the effect of age differed between boys and girls. Model 3b estimated the interaction between age*immigrant background to test whether the effect of age differed between children of immigrants and nonimmigrants. Model 3c included both the interaction age*gender and age*immigrant background. Model 3d, finally, included the 3-way interaction age*gender*immigrant background to examine whether the interaction between age and immigrant background differed between boys and girls. Likelihood ratio(LR)-tests and BIC’s were used to compare models. Lower BIC’s indicate a better model fit. To interpret the regression models, main effects (from models 2a–2d) are presented in Table 2. The models with age interactions (models 3a–3d) and their predicted margins and confidence intervals are presented graphically (Figs. 2–3).

**Table 2:** Selected random effects models of life satisfaction

Independent variable	Model 1b	Model 2a	Model 2b	Model 2c	Model 2d
Girl (vs. boy)		−0.378^*^ (0.000)		−0.374^*^ (0.000)	−0.319^*^ (0.000)
Immigrant background (vs. nonimmigrant)			−0.136^*^ (0.004)	−0.115^*^ (0.015)	0.018 (0.796)
Girl * immigrant background					−0.245^*^ (0.009)
Age 14 (reference)					
Age 15	−0.24 (0.626)	−0.031 (0.534)	−0.023 (0.646)	−0.030 (0.551)	−0.030 (0.543)
Age 16	0.176^*^ (0.001)	0.167^*^ (0.001)	0.178^*^ (0.000)	0.169^*^ (0.001)	0.169^*^ (0.001)
Age 17	0.179^*^ (0.000)	0.176^*^ (0.000)	0.180^*^ (0.000)	0.177^*^ (0.000)	0.177^*^ (0.000)
Age 18	−0.047 (0.368)	−0.047 (0.360)	−0.046 (0.373)	−0.047 (0.365)	−0.047 (0.368)
Age 19	0.106^*^ (0.049)	0.105 (0.052)	0.107^*^ (0.048)	0.105 (0.051)	0.106 (0.050)
Age 20	0.284^*^ (0.000)	0.284^*^ (0.000)	0.284^*^ (0.000)	0.284^*^ (0.000)	0.283^*^ (0.000)
Age 21	0.016 (0.819)	0.020 (0.771)	0.016 (0.816)	0.020 (0.769)	0.021 (0.764)
Age 22	−0.172^*^ (0.006)	−0.170^*^ (0.006)	−0.172^*^ (0.006)	−0.169^*^ (0.006)	−0.168^*^ (0.007)
Age 23	−0.234^*^ (0.035)	−0.245^*^ (0.027)	−0.231^*^ (0.037)	−0.242^*^ (0.028)	−0.243^*^ (0.028)
Constant	7.521^*^ (0.000)	7.718^*^ (0.000)	7.551^*^ (0.000)	7.740^*^ (0.000)	7.713^*^ (0.000)
σ_u_	1.016^*^ (0.000)	0.997^*^ (0.000)	1.013^*^ (0.000)	0.995^*^ (0.000)	0.994^*^ (0.000)
σ_e_	1.670^*^ (0.000)	1.670^*^ (0.000)	1.670^*^ (0.000)	1.670^*^ (0.000)	1.670^*^ (0.000)
Rho	0.263	0.263	0.269	0.262	0.262

*Note*: σ_u_ = Between-person standard deviation, σ_e_ = within-person standard deviation *p*-values in parentheses, *p < 0.05

Finally, sensitivity analyses were performed. To investigate attrition effects, main analyses were performed on a restricted sample of participants who participated in all seven waves. Differences within the group of children of immigrants were explored based on their country of origin and religion.

## Results

Model comparison results of the hierarchical multilevel models are presented in Table 3. Model 0 shows that both within-person (σ_e_ = 1.68, *SE* = 0.01) and between-person standard deviations (σ_u_ = 1.01, *SE* = 0.18) were significant. The intraclass correlation (ICC) indicates that 26.8% of the variance was between persons and 73.2% within persons. In other words, there was a strong correlation between individuals’ life satisfaction levels over time (*r* = 0.73).

**Table 3:** Model hierarchy and comparisons of nested models of life satisfaction

Models		BIC	Chi^2^(*df*)
Model 0 =	Empty	88538.77	
Model 1a =	Model 0 + Age linear	88517.17	41.31(2)**
Model 1b =	Model 0 + Age discrete	88505.40	122.06(9)**
Model 2a =	Model 1b + gender	88424.21	91.05(1)**
Model 2b =	Model 1b + immigrant background	88507.07	8.19(1)**
Model 2c =	Model 2a + immigrant background	88428.12	5.94(1)*
Model 2d =	Model 2c + gender * immigrant background	88431.24	6.74(1)**
Model 3a =	Model 2a + gender * age	88482.71	30.19(9)**
Model 3b =	Model 2b + migration * age	88587.37	8.39(9)
Model 3c =	Model 3a + immigrant background * age	88565.80	15.45(10)
Model 3d =	Model 3c + gender * migration * age	88638.00	26.35(10)**

*Note*: Models with interactions always include the main effects. *N* observations = 19,041, *N* participants = 3757

*p < 0.05, **p < 0.01

**Fig. 1:**
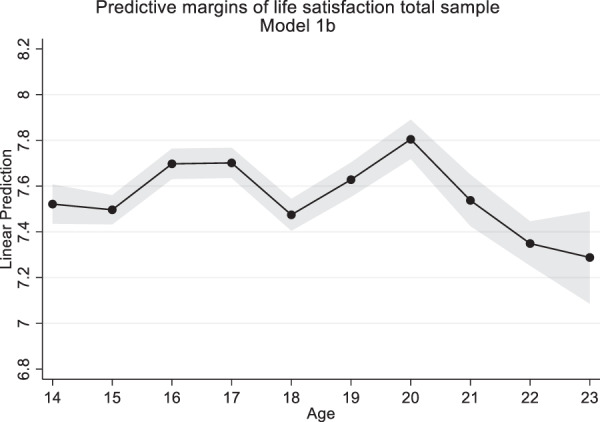
Predicted margins of life satisfaction with 95% confidence intervals

In Model 1a, the predictor age was added. Both the linear (*b* = 0.45, *SE* = 0.07, *p* < 0.001) and quadratic (*b* = −0.01, *SE* = 0.002, *p* < 0.001) term were significant. Model fit improved significantly (*χ*^2^(2) = 41.31, *p* < 0.001) compared to the empty model indicating the importance of age and thus development over time (Table 2). To examine in more detail how life satisfaction developed over time, Model 1b included age as an indicator variable. Fit of Model 1b improved compared to Model 1a, indicating a nonlinear age pattern (*χ*^2^(7) = 80.75, *p* < 0.001). Life satisfaction means fluctuated but remained above seven over the entire study length. In contrast to the expected U-shape, life satisfaction followed an M-shape: it increased until age 16, then decreased until age 18, increased until age 20, but again decreased until age 23 (Fig. 1). This suggests a nonlinear development of life satisfaction over time. Because of the nonlinear development of life satisfaction, age was included as an indicator variable in the models with interactions by gender and immigrant status.

In random intercepts Model 2a, gender was added to examine whether *levels* of life satisfaction differed between boys and girls (Table 3). Model fit improved compared to Model 1b (*χ*^2^(1) = 91.05, *p* < 0.001). As expected, life satisfaction levels were lower for girls (*b*_*girl*_ = −0.39, *SE* = 0.04, *p* < 0.001). The effect size was *d* = 0.39/1.51 = 0.26 which was a small to medium effect (Cohen 1988). In Model 2b, immigrant background was included. Model fit improved compared to Model 1b (*χ*^2^(1) = 8.19, *p* < 0.001). Children of immigrants had lower life satisfaction levels than nonmigrants (*b*_immigrant background_ = −0.14, *SE* = 0.05, *p* = 0.015), but the difference was small in magnitude (Cohen’s *d* = 0.09). Model 2c included both gender and immigrant background. Model fit improved slightly compared to Model 2a (*χ*^2^(1) = 5.94, *p* = 0.015), indicating that immigrant background significantly added to the explanation of differences in levels of life satisfaction above and beyond gender, (*b*_immigrant background_ = −0.12, *SE* = 0.05, *p* = 0.015), although this addition was negligible in magnitude (Cohen’s *d* = 0.08). Model 2d included the interaction gender*immigrant background. Model fit improved (*χ*^2^(1) = 6.74, *p* = 0.009) compared to Model 2c and the interaction was negative and significant (*b*_girl*immigrant background_ = −0.25, *SE* = 0.09, *p* = 0.009, Cohen’s *d* = 0.17). Interpreted together with the main effect of immigrant background (which applies to boys) the findings show that having an immigrant background had a small but negative effect on life satisfaction for girls, but not for boys. Thus, girls with immigrant backgrounds had the lowest levels of life satisfaction, supporting the double disadvantage hypothesis.

**Fig. 2:**
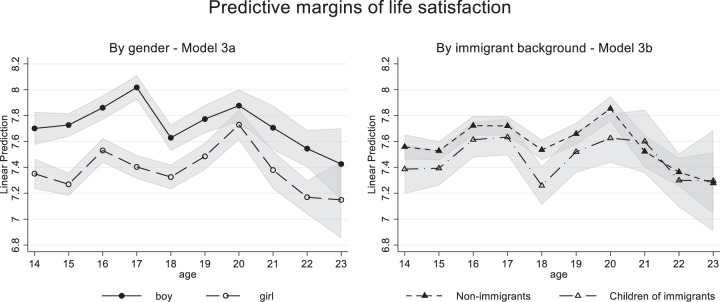
Predicted margins of cross-level interaction models with 95% confidence intervals

In Model 3a, the cross-level interaction age*gender was estimated to examine whether *development* differed between boys and girls. Model 3a improved the fit compared to Model 2a, which only allowed for gender differences in levels of life satisfaction (*χ*^2^(9) = 30.19, *p* < 0.001), indicating significant differences in life satisfaction trajectories between boys and girls. In Fig. 2, predicted margins and their confidence intervals are presented by age and gender. Boys’ life satisfaction increased between ages 15 and 17, dropped at age 18, increased slightly to age 20, and then decreased to age 23. Girls’ life satisfaction increased between age 15 and 16, decreased slightly up to age 18, increased to age 20, but decreased again to age 23. As expected from the accelerated maturation hypothesis and the stress-sensitivity perspective, the gender gap in life satisfaction levels decreased with age and was no longer significant at ages 20 and 23.

Model 3b tested the cross-level interaction age*immigrant background. Model 3b did not significantly improve model fit compared to Model 2b (χ^2^(9) = 8.39, *p* = 0.495), indicating similar development for children of immigrants and nonimmigrants (Fig. 2). Model 3c combined the two two-way interactions age*gender and age*immigrant background. Adding immigrant background did not add significantly above and beyond gender (χ^2^(10) = 15.45, *p* = 0.116). Figure 2 demonstrates similar trajectories for children of immigrants and nonimmigrants, except for a steeper drop at age 18 for children of immigrants.

**Fig. 3:**
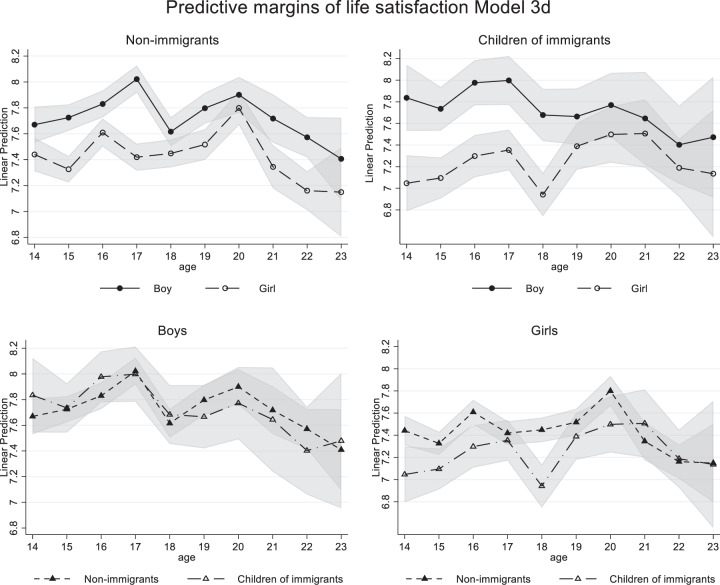
Predicted margins of Model 3d by gender (top panel) and by immigrant background (bottom panel) with 95% confidence intervals

The final Model 3d tested a three-way interaction age*gender*immigrant background, which significantly improved model fit compared to Model 3c, which only contained the two two-way age-interactions (χ^2^(10) = 26.35, *p* = 0.003). Figure 3 shows the results of Model 3d but splits the lines for boys and girls and children of immigrants and nonimmigrants into separate figures for better visibility.

For nonimmigrant adolescents, both levels and trajectories of life satisfaction differed by gender but differences decreased from age 18 onwards and were no longer significant at age 20 and 23. For children of immigrants, strong gender differences in levels were found in adolescence, but the gender gap disappeared completely from age 18 onward, resulting in similar life satisfaction levels and development in emerging adulthood. Boys with and without immigrant backgrounds showed similar trajectories of life satisfaction, with a drop at age 18, an increase until age 20, and a decrease until age 23. However, girls with and without immigrant backgrounds had different trajectories. Girls with immigrant backgrounds started with the lowest life satisfaction at age 14, increased slightly until age 16, reported a steep fall at age 18 but showed the strongest increase of the four groups from age 18 to 20, and decreased again until age 23. Nonimmigrant girls did not report the remarkable dip at 18 but showed a decreasing trajectory from age 20 to 23. In short, there are gender differences in life satisfaction trajectories, but primarily for children of immigrants.

Because the group of children of immigrants is heterogeneous in terms of origin country and religion, this study explored life satisfaction trajectories for the five largest children of immigrant groups separately (i.e., from Turkey, Former Soviet Union, Poland, Former Yugoslavia, and Italy). Moreover, since 51.8% of all children of immigrants in our study identified as Muslim, trajectories for Muslim and non-Muslim children of immigrants were explored separately. Overall, trajectories did not differ significantly between groups. The gender gap and dip in life satisfaction for girls at age 18 was robust for most countries of origin and for Muslim and non-Muslim children of immigrants (full details available on request of the first author).

### Sensitivity Analyses

To examine the effect of attrition on the results, sensitivity analyses were performed with a restricted sample of participants who participated in all 7 waves (see Appendix, Fig. A2). Overall, findings were robust: the same M-shaped pattern of life satisfaction was found in the restricted sample, where girls reported lower levels than boys. Again, girls with immigrant backgrounds had the lowest levels across adolescence, with a significant dip at age 18. As in the full sample, differences diminished in emerging adulthood. As attrition analyses (Appendix 1, Table A1) and wave-specific dropout rates (Appendix 1, Table A2) showed, immigrant boys with higher life satisfaction were somewhat more likely to drop out. This means that the results of the full sample could be underestimating life satisfaction for boys with immigrant backgrounds. However, the sensitivity analyses showed that this effect was especially visible at age 18. That is, the boys with immigrant backgrounds from the restricted sample showed a more stable decreasing pattern without the fall at age 18. Importantly, their relative position compared to the other groups remained the same as in the full sample, which means that the conclusions of this study were not fundamentally affected by selective attrition.

## Discussion

Given the importance of life satisfaction for positive development in the transition from adolescence to adulthood, it is essential to know how life satisfaction develops over this transition period. The limited previous research on life satisfaction development had focused on either adolescence or adulthood. Moreover, research into diversity in trajectories for boys and girls and those with and without immigrant backgrounds was largely absent. More insight into life satisfaction development in the transition to adulthood for different subgroups is important to pinpoint subgroups at risk in diverse societies and to identify the critical turning points in life satisfaction development. This may help to understand the evolvement of the individual life course, inequalities, and develop adequate interventions. The seven-wave longitudinal data used in this study provide unique opportunities to fill these gaps in the literature. Results showed first of all that, on average, the young people in this study had relatively high levels of life satisfaction but that levels changed in the transition to adulthood. Secondly, overall, girls had lower levels and more pronounced ups and downs than boys. This was especially accentuated for girls with immigrant backgrounds. Third, while for nonimmigrants gender differences decreased in emerging adulthood, for children of immigrants these differences disappeared completely after age 18. Fourth, age 18 seemed to be a key turning point for all when life satisfaction fell remarkably. Nonimmigrant girls were the only exception to this.

### General M-Shaped Trajectory

Following the stage theory of psychosocial development (Erikson, 1968), theory of emerging adulthood (Arnett, 2000), and unified theory of human development (Sameroff, 2010), it was expected that life satisfaction would decrease due to the turbulence in the transition to adulthood but would increase again when young people navigated through the transition successfully. Contrary to the U-shape expected based on these theories, results showed an M-shaped development of life satisfaction, with a dip at age 18 and a decrease after age 20. It should be noted that the average fluctuations in the total sample were modest due to individual variability. In light of the individualization of the life course in Western societies, where the transition to adulthood has been extended (Liefbroer & Toulemon, 2010), this M-shape may be less surprising than originally expected. The major life events related to life satisfaction (e.g., leaving the parental home, entering the labor market, or starting a family) are not concentrated at one specific age but are spread out over the entire transition to adulthood (Kins & Beyers, 2010; Switek & Easterlin, 2018). The M-shaped pattern may thus be composed of consecutive U-shapes, in which the dips may relate to different major life transitions. For example, the decrease in life satisfaction between ages 16 and 18 could be linked to the end of secondary education (Kristen & Granato, 2007) while the decrease after age 20 might be linked to school-to-work transitions (Creed et al., 2003). These transitions are potentially stressful as they are often accompanied by, for example, new responsibilities, changes in social networks, and residential mobility. This might lead to temporary lower levels of life satisfaction, followed by recovery when young people get used to the new situation and find equilibrium again. It indicates that vulnerabilities arise especially around these key transitions, which may result in long-term inequalities in young people’s well-being. Similarly, the increases in life satisfaction between ages 18 and 19 could be related to the fact that young people are legally adults from age 18 onwards. The increased freedom, opportunities, and independence that come with turning 18 may increase life satisfaction.

That this study found a decrease instead of the expected increase into emerging adulthood might indicate that not the entire transition to adulthood was captured for all participants. That is, at age 23, most emerging adults in Europe have not gone through all transitions in the public and private domain of life (including for example finishing education, leaving the parental home, finding a job, or marriage; Eurostat, 2017, 2018, 2019). Future research would benefit from extending this research, by aiming for an even longer longitudinal follow-up. Although this work points to some of the potential factors explaining life satisfaction development, future research, including qualitative research, is needed to study underlying mechanisms in more detail.

### Subgroup Differences

Based on gender differences in human development, different developmental patterns of life satisfaction for boys and girls were expected. Although an M-shape was visible for both boys and girls, clear gender differences were found as well. Overall, girls had lower life satisfaction levels and more pronounced ups and downs than boys. After age 18 these differences in trajectories diminished but lower levels for girls remained. A heightened sensitivity for stress in adolescent girls could explain these findings. That is, gender differences in stress sensitivity may increase under the influence of hormonal changes from puberty onwards and decrease again into adulthood, which makes girls in general more sensitive to stressful life events, especially in adolescence (see Oldehinkel & Bouma, 2011 for a review). Moreover, girls are generally more responsive to interpersonal stressors, such as those related to peer relations or family conflict (Kendler et al., 2001). These stressors typically increase during adolescence, which could negatively affect life satisfaction especially among adolescent girls (De Looze et al., 2020; Yucel & Yuan, 2016). In addition, in line with the accelerated maturation hypothesis, gender differences in this study were clear in adolescence but disappeared in emerging adulthood. This may explain why adolescent research consistently found girls to report lower life satisfaction (Kaman et al., 2020), while adult research found women to score higher (Orben et al., 2020). These findings highlight the importance of going beyond cross-sectional measurement and studying life satisfaction longitudinally, especially in a life phase where many transitions come together in a short period of time.

Furthermore, results were in line with the cultural mismatch perspective for girls with immigrant backgrounds. As expected based on the double disadvantage hypothesis (King, 1988; Ghavami et al., 2016), adolescent girls with immigrant backgrounds had lower life satisfaction levels than nonimmigrant girls and boys with immigrant backgrounds, with a remarkable dip at age 18. Gendered socialization pressures may explain why especially adolescent girls with immigrant backgrounds struggled to maintain high life satisfaction in the transition to adulthood (Dion & Dion, 2001). Traditionally gendered values in immigrant families are marked by greater restrictiveness and monitoring for adolescent girls with immigrant backgrounds compared to their male counterparts (Idema & Phalet, 2007; Suárez‐Orozco & Qin, 2006). In addition, the pressure to behave according to traditional values in immigrant families is greater for girls than for boys (Dion & Dion, 2001). As such, adolescent girls with immigrant backgrounds may enjoy less freedom than boys with immigrant backgrounds, but also than girls without immigrant backgrounds. Also, they may feel “trapped between competing values, such as between the need for personal development and the need to maintain family traditions and secure family relationships” (Dion & Dion, 2001, p. 518). This cultural mismatch might be especially prominent in adolescence (Céspedes & Huey, 2008). From the age of 18, all girls in Germany are legally adults and there is more room for making one’s own decisions. For girls with immigrant backgrounds, the influence of gendered socialization pressures from parents may diminish from age 18 onward and thus becoming of age may make more difference for girls with immigrant backgrounds than for groups that already enjoyed more freedom in adolescence (i.e., girls without immigrant backgrounds, boys with and without immigrant backgrounds). This could explain why life satisfaction of girls with immigrant backgrounds increased more in emerging adulthood than in any other group.

In line with the concept of emerging adulthood (Arnett, 2000, Arnett, 2007), differences between groups decreased after adolescence. Emerging adulthood is the phase in life with most demographic variability (Arnett, 2000). This could be linked to the de-standardization, or individualization, of young people’s pathways into adulthood in Western societies (Beck & Beck-Gernsheim, 2002). Due to this de-standardization of the life course, there is more room for young people to make their own choices in life instead of following standardized life-course trajectories (e.g., mandatory secondary education, standardized sequencing of marriage, cohabitation, and parenthood; Liefbroer & Toulemon, 2010). This de-standardization may decrease patterns in which life satisfaction trajectories are based on structured groupings, as by gender or immigrant background, and as such may diminish between-group differences in life satisfaction.

### Limitations

The longitudinal design of this study and its diverse sample allowed to study life satisfaction development in different subgroups, which is a unique asset that has been largely absent in the literature until now. However, some limitations of the data should be addressed. First, although robustness checks did not reveal large effects of panel attrition, attrition analyses indicated that higher life satisfaction in the previous wave was a weak but significant predictor of dropout in the next wave for boys with immigrant backgrounds. Sensitivity analyses showed that life satisfaction levels of immigrant boys were potentially somewhat underestimated, especially at age 18. Second, since the first three waves were conducted in schools, the study sample could be selective with regard to school dropouts. Third, given the extended transition to adulthood of the last decades (Liefbroer & Toulemon, 2010), this study probably did not capture the entire transition to adulthood for all participants. Future research should aim for an even longer longitudinal follow-up. Fourth, since data was structured by age (and included all participants aged 14–16 at wave 1), the wave structure and therewith the cohort effects were lost. Thus, participants for example could have been 16 in 2010, 2011, or 2012 (i.e., wave 1, 2, or 3). It was therefore impossible to disentangle between age, cohort, and potential period effects. More advanced data on large samples are needed to go beyond this issue in future studies. Finally, the results of this study might be specific for young people who are living in Germany or, more general, in individualistic Western European cultures and may not be generalizable to young people in other social contexts.

## Conclusion

So far, it was largely unknown how life satisfaction developed in the transition to adulthood and whether developmental trajectories differed by gender and immigrant background. This study used longitudinal data on a large population sample of young people between the ages 14 and 23 living in Germany to bridge the gap between adolescent and adult life satisfaction research. Results showed general M-shaped fluctuations in life satisfaction over the transition to adulthood, with a dip at age 18. However, differences in developmental trajectories between subgroups were found. Compared to boys, girls, and girls with immigrant backgrounds in particular, reported larger ups and downs and lower levels of life satisfaction in adolescence. Subgroup differences disappeared in emerging adulthood. This study suggests some implications for future research. First, these results highlight the importance of longitudinal research. That is, cross-sectional research can only provide snapshots of life satisfaction differences and depending on the age that is measured, results could provide a different picture. Extrapolating results beyond the age of measurement could therefore result in incorrect conclusions. Future longitudinal research should follow participants even longer to investigate how gender and immigrant background differences evolve with aging. Second, gender and immigrant background are found to be essential when investigating life satisfaction in the future, especially in adolescent research. Third, future research is encouraged to investigate to what extent life events in the transition to adulthood can explain the fluctuations in life satisfaction and what factors contribute to the differences between social groups. In addition, this study suggests some implications for practitioners and policymakers. First, because life satisfaction in the transition to adulthood fluctuates, it could be effective to already intervene in adolescence and emerging adulthood and as such prevent inequalities with potentially long-lasting effects. Second, the diversity in life satisfaction trajectories in adolescence indicates that interventions may be more effective when tailored to the different subgroups. The accumulation of disadvantages may put some young adults in more vulnerable positions, in this case girls with immigrant backgrounds. Special attention is needed for those situations in which inequalities accumulate already at a young age, as this may be perpetuated and accentuated later in life and influence individual life chances over the life course.

## Supplementary information

Appendix A1

## Footnotes

1 In this study, the term young people is used to refer to both middle/late adolescents (age 14–17) and emerging adults (age 18–23)

2 Although waves 1 to 6 were aimed to follow up with 12-month intervals (waves 6 and 7 were aimed 2 years apart), not all consecutive waves were exactly 12 months apart (ranging between 10 months to in some extreme cases 20 months between 2 waves). This resulted in some participants (number of relevant cases ranging between *n* = 199 and *n* = 1) being the same age in two consecutive waves or skipping one age. Because age effects were of primary interest, it was considered undesirable to ‘smooth’ the age changes during the panel. Therefore, it was decided to not adjust participants’ ages.
