# Supplementary material for: Life Satisfaction Development in the Transition to Adulthood: Differences by Gender and Immigrant Background
Source: J Youth Adolesc. 2022 Jan 13;51(2):305–19. doi: 10.1007/s10964-021-01560-7 (PMC8828595; doi:10.1007/s10964-021-01560-7)
Supplement: Supplementary file 1 — Appendix A1 [file 10964_2021_1560_MOESM1_ESM.docx]

**Appendix A1**

Supplemental Material

This Appendix contains supplemental material, including attrition analyses, wave-specific dropout statistics, and the figure-output of the sensitivity analyses of Model 3d.

Table A1

*Weighted Logistic Regression Predicting Attrition by Gender, Immigrant Background, and Life Satisfaction*

|  | **Drop out** |
| --- | --- |
| Age | .176^*^ (.000) |
| Children of immigrants | -.667 (.139) |
| Girl | -.114 (.738) |
| Imm back * girl | .751 (.203) |
| Life satisfaction | -.008 (.802) |
| Life satisfaction * girl | -.031 (.479) |
| Life satisfaction * imm back | .131^*^ (.022) |
| Life satisfaction * imm back * girl | -.118 (.124) |
| Constant | -4.829^*^ (.000) |
| *N* | 17311 |

*Note.* CILS4EU wave 1-7 (2010-2018) in Germany.
P-values in parentheses, * *p* < .05. Imm back = Children of immigrants

Table A2

*Unweighted Dropout Statistics per Wave for Different Subgroups*

|  | **Wave** | | | | | |
| --- | --- | --- | --- | --- | --- | --- |
|  | **T = 1** | **T = 2** | **T = 3** | **T = 4** | **T = 5** | **T = 6** |
| Non-immigrant boys |  |  |  |  |  |  |
| Dropout percentage between wave T and T+1 | 2.4 | 23.3 | 14.6 | 12.3 | 21.0 | 19.0 |
| Mean LS difference^a^ (*SE*) | -0.1 (0.42) | 0.1 (0.15) | 0.01 (0.16) | -0.3 (0.21) | 0.02 (0.18) | -0.1 (0.15) |
| Boys with immigrant backgrounds |  |  |  |  |  |  |
| Dropout percentage between wave T and T+1 | 4.9 | 29.3 | 20.0 | 16.4 | 34.4 | 20.6 |
| Mean LS difference (*SD*) | -0.3 (0.37) | -0.2 (0.19) | -0.2 (0.19) | -0.5 (0.27) | 0.3 (0.23) | 0.4 (0.23) |
| Non-immigrant girls |  |  |  |  |  |  |
| Dropout percentage between wave T and T+1 | 4.1 | 16.8 | 12.0 | 6.3 | 21.3 | 10.8 |
| Mean LS difference (*SD*) | 0.2 (0.33) | 0.1 (0.18) | 0.2 (0.17) | -0.1 (0.28) | 0.3 (0.16) | -0.3 (0.17) |
| Girls with immigrant backgrounds |  |  |  |  |  |  |
| Dropout percentage between wave T and T+1 | 4.5 | 16.9 | 13.8 | 7.2 | 22.2 | 17.5 |
| Mean LS difference (*SD*) | -0.3 (0.41) | 0.4 (0.24) | 0.1 (0.2) | -0.2 (0.37) | 0.3 (0.22) | 0.1 (0.21) |

*Note.* ^a^ Difference between life satisfaction (LS) of participants participated in the next wave and those who did not participate in the next wave.

**Fig. A1** Predicted margins of final Model 3d in restricted sample of participants participated in all seven waves. Life satisfaction levels over the transition to adulthood for children of immigrants and non-immigrants by gender, with 95% confidence intervals
